# Supplementary material for: Maize variety preferences among smallholder farmers in Ethiopia: Implications for demand-led breeding and seed sector development
Source: PLoS One. 2022 Sep 29;17(9):e0274262. doi: 10.1371/journal.pone.0274262 (PMC9522265; doi:10.1371/journal.pone.0274262)
Supplement: S1 File — (DOCX) [file pone.0274262.s001.docx]

**Appendix 1: The Choice Experiment Process**

After a detailed explanation of the difference between traits in the two varieties in question, the respondent is asked to choose one preferred variety i.e., either variety one (*Gosa-sanyi 1*) or variety two (*Gosa-sanyi 2*). In **Session 1 (Experiment C)** below for instance (Fig. A1), **variety one** has a maximum yield of 10 quintals/*timad*, it is not sweet when roasted or boiled and, while **variety two** has also has maximum 10 quintals/*timad*, it is sweet when roasted or boiled and, it has poor standability (lodges easily). Each respondent was made to select one preferred variety in 12 sessions, similar to the one in fig. A1. To test whether the respondents truly understood the question and before making a final selection in any experimental session, the interviewers were instructed to allow the respondent a minimum of 20-30 seconds to evaluate the choice before settling on the preferred alternative. More time was allowed for the respondent to ask questions. The combination of un-rushed choice process and the chance to ask clarifying questions, limiting the choices only to pairwise comparisons, preliminary explanation in each session and use of vivid pictures ensured there was low cognitive burden on the respondents and comprehension was near-guaranteed in almost all cases. The descriptions were administered in local Oromo language.


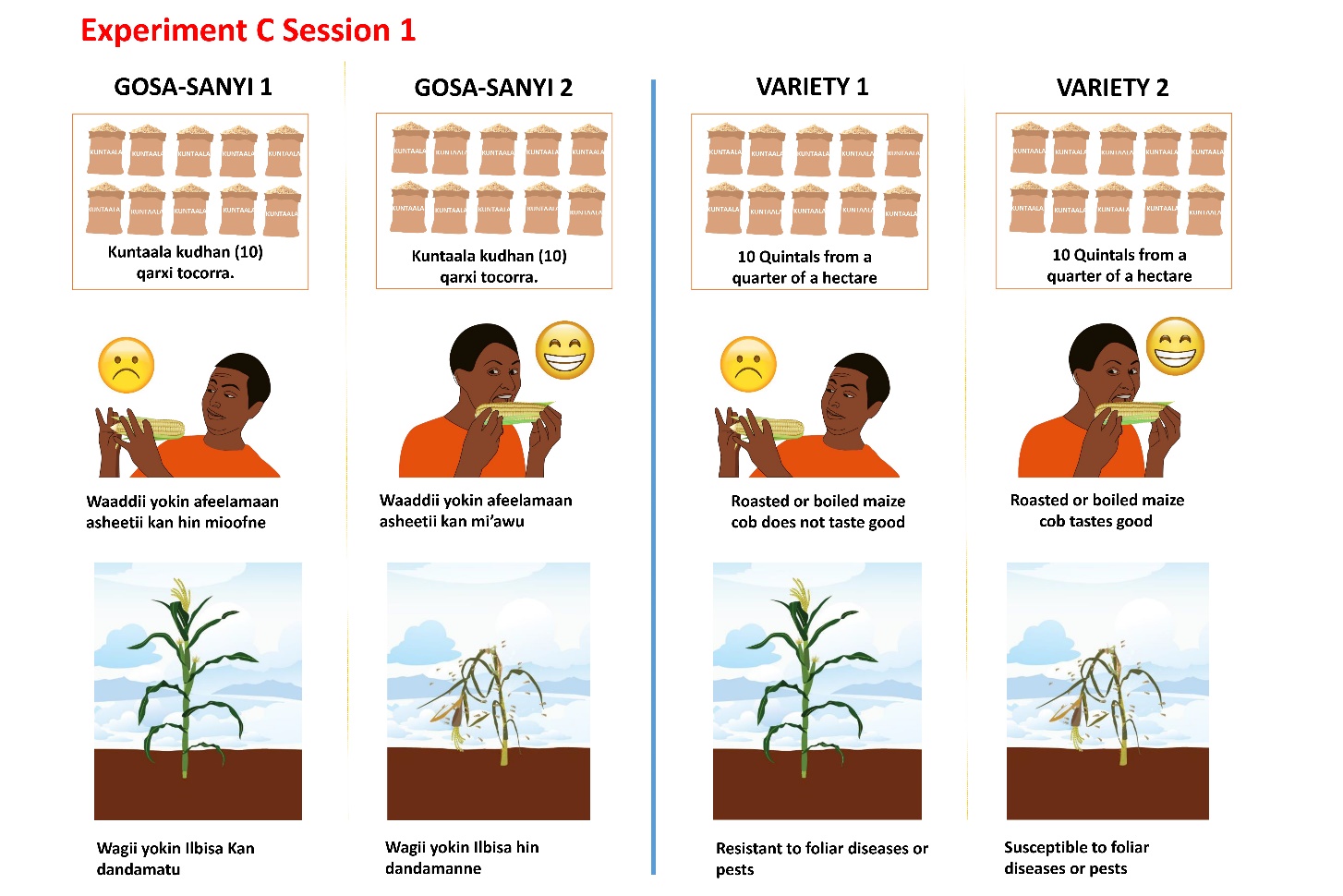


**Fig. A1:** Example of a choice experiment session (Experiment C, Session 1 in this case). The one at left is Oromia language, widely spoken in the study communities. At bottom is the English translation

**Appendix 2:**

**The BDM Process**

Similar to the CEs, the BDMs involved presenting a series of thought experiments (auctions) to farmers to choose between pairs of varieties. One trait was used to compare two varieties at a time. To illustrate: regarding open or closed tip variety, one variety was described as open tip and the other closed tip. The farmer was taken through a series of comparisons of the yields between the two varieties starting with first step where both varieties are deemed to yield the same amount. The yield of the variety with the desirable trait (closed tip in this case) was lowered gradually while maintaining the yield of the variety with the less desirable trait). Each time the farmer was asked to choose which variety they would prefer. The point at which the farmer chose to switch to the variety with the less desirable trait was deemed to be their maximum wiliness to sacrifice yield (WTSY) for the desirable trait. The starting point was 7 quintals per *timad* for both varieties. If the farmer switched to the open tip variety when the yield of the closed tip variety was 5 quintals/*timad*, the WTSY for the closed tip variety was calculated as 2 quintals/*timad* (7 minus 5 quintals). The process was made incentive compatible (giving respondents incentive to reveal their true WTSY) as per [38]. The actual procedure for the BDM is described *verbatim* below, illustrated by the BDM session for closed tip. Since the BDM was simpler the participants did six rounds, one for each trait (with yield being the “price”).

**Verbatim example of the BDM experiment**

*In what follows, I would like to ask you how much you prefer two varieties. You will notice that one of the varieties has the desirable trait, the other variety does not have the desirable trait. We will start by comparing the two varieties when the yields are the same. Then after that, you will state how much the yield of the good trait variety can go down before you decide to choose the variety which doesn’t have the good trait. When you* ***reach*** *this point (After you switch from your original choice to the variety which does not have the good trait) we will draw from a secret list the yields that range from 0.5 quintals per timad to 7 quintals per timad. If you pick a yield that is* ***equal or more than*** *the yield you stated, you will earn points from this yield (1 quintal=1 point). At the end of the game 1 point=6 birr. If you pick a yield that is* ***lower*** *than what you stated, you will earn* ***zero*** *points. Therefore, is in your best interest to state the accurate yield reduction that you would be willing tolerate in real life. For example, if you state too low compared to the true yield you would be willing to sacrifice, you are likely to earn only few points even if you pick the one greater than your choice. If you state too high than what you would sacrifice in real life, you might end up losing the game, because many of the yields will fall below your choice. To repeat, it is better to say what you really think is the true yield you would sacrifice for the good variety in real life. [The example below is session on husk cover (session 5)].*

*I would like to ask you about the following two maize varieties, for one the tip or husk cover is open, and the other tip or husk cover is closed:*

| Suppose the one that has **TIP OR HUSK COVER OPEN** yielded  **7** quintals per timad | AND the one that has T**IP OR HUSK COVER CLOSED yielded**  **7** quintals per timad | Which would you choose? |
| --- | --- | --- |
| Suppose the one that has **TIP OR HUSK COVER OPEN** yielded  **7** quintals per timad | AND the one that has T**IP OR HUSK COVER CLOSED yielded**  **6.5** quintals per timad | Which would you choose? |
| Suppose the one that has **TIP OR HUSK COVER OPEN** yielded  **7** quintals per timad | AND the one that has T**IP OR HUSK COVER CLOSED** yielded  **6** quintals per timad | Which would you choose? |
| … | **…** | … |
| Suppose the one that has **TIP OR HUSK COVER OPEN** yielded  **7** quintals per timad | AND the one that has T**IP OR HUSK COVER CLOSED** yielded  **5** quintals per timad | Which would you choose? |
| Suppose the one that has **TIP OR HUSK COVER OPEN** yielded  **7** quintals per timad | AND the one that has T**IP OR HUSK COVER CLOSED** yielded  **4.5** quintals per timad | Which would you choose? |
| Suppose the one that has **TIP OR HUSK COVER OPEN** yielded  **7** quintals per timad | AND the one that has T**IP OR HUSK COVER CLOSED** yielded  **4** quintals per timad | Which would you choose? |
| Suppose the one that has **TIP OR HUSK COVER OPEN** yielded  **7** quintals per timad | AND the one that has T**IP OR HUSK COVER CLOSED** yielded  **3.5** quintals per timad | Which would you choose? |
| Suppose the one that has **TIP OR HUSK COVER OPEN** yielded  **7** quintals per timad | AND the one that has T**IP OR HUSK COVER CLOSED** yielded  **3** quintals per timad | Which would you choose? |
| Suppose the one that has **TIP OR HUSK COVER OPEN** yielded  **7** quintals per timad | AND the one that has T**IP OR HUSK COVER CLOSED** yielded  **2.5** quintals per timad | Which would you choose? |
| Suppose the one that has **TIP OR HUSK COVER OPEN** yielded  **7** quintals per timad | AND the one that has T**IP OR HUSK COVER CLOSED** yielded  **2** quintals per timad | Which would you choose? |
| Suppose the one that has **TIP OR HUSK COVER OPEN** yielded  **7** quintals per timad | AND the one that has T**IP OR HUSK COVER CLOSED** yielded  **1.5** quintals per timad | Which would you choose? |
| Suppose the one that has **TIP OR HUSK COVER OPEN** yielded  **7** quintals per timad | AND the one that has T**IP OR HUSK COVER CLOSED** yielded  **1** quintals per timad | Which would you choose? |
| Suppose the one that has **TIP OR HUSK COVER OPEN** yielded  **7** quintals per timad | AND the one that has T**IP OR HUSK COVER CLOSED** yielded  **0.5** quintals per timad | Which would you choose? |

**Appendix 3**

**Table A3: Mixed logit results on willingness to pay for traits (without interactions)**

| Variables | Pooled | |  | Male respondent | |  | Female respondent | |
| --- | --- | --- | --- | --- | --- | --- | --- | --- |
|  | Coeff | SD |  | Coeff | SD |  | Coeff | SD |
| **Experiment A** |  |  |  |  |  |  |  |  |
| Yield | 1.671*** |  |  | 1.698*** |  |  | 1.676*** |  |
|  | (0.069) |  |  | (0.099) |  |  | (0.097) |  |
| Drought tolerant | 4.740*** | 0.766*** |  | 4.845*** | 0.790*** |  | 4.724*** | 0.791*** |
|  | (0.160) | (0.074) |  | (0.236) | (0.116) |  | (0.227) | (0.103) |
| Matures in 3 months or less | -0.487*** | -0.052 |  | -0.519*** | 0.290** |  | -0.479*** | 0.112 |
|  | (0.048) | (0.320) |  | (0.073) | (0.129) |  | (0.067) | (0.209) |
|  |  |  |  |  |  |  |  |  |
| Observations | 9,000 | 9,000 |  | 4,464 | 4,464 |  | 4,536 | 4,536 |
| chi2 | 65.50 | 65.50 |  | 26.60 | 26.60 |  | 38.15 | 38.15 |
| Log Likelihood | -1877 | -1877 |  | -923.6 | -923.6 |  | -953.5 | -953.5 |
| **Experiment B** |  |  |  |  |  |  |  |  |
| Yield | -0.059** |  |  | -0.109** |  |  | -0.017 |  |
|  | (0.029) |  |  | (0.043) |  |  | (0.040) |  |
| Lodging resistant | 0.277*** | 0.800*** |  | 0.159* | 0.798*** |  | 0.375*** | 0.878*** |
|  | (0.066) | (0.079) |  | (0.094) | (0.113) |  | (0.094) | (0.110) |
| Closed tip | 1.044*** | 1.156*** |  | 1.182*** | 1.206*** |  | 0.978*** | 1.110*** |
|  | (0.074) | (0.068) |  | (0.108) | (0.096) |  | (0.100) | (0.093) |
|  |  |  |  |  |  |  |  |  |
| Observations | 8,808 | 8,808 |  | 4,344 | 4,344 |  | 4,464 | 4,464 |
| chi2 | 442.3 | 442.3 |  | 234.5 | 234.5 |  | 215.6 | 215.6 |
| Log Likelihood | -2454 | -2454 |  | -1167 | -1167 |  | -1277 | -1277 |
| **Experiment C** |  |  |  |  |  |  |  |  |
| Yield | 0.711*** |  |  | 0.703*** |  |  | 0.711*** |  |
|  | (0.046) |  |  | (0.067) |  |  | (0.063) |  |
| Sweet taste | 2.013*** | -1.180*** |  | 2.190*** | 1.111*** |  | 1.830*** | 1.177*** |
|  | (0.105) | (0.108) |  | (0.156) | (0.140) |  | (0.152) | (0.154) |
| Rust resistant | 5.061*** | 3.003*** |  | 5.205*** | 3.234*** |  | 4.586*** | 2.614*** |
|  | (0.259) | (0.221) |  | (0.383) | (0.323) |  | (0.313) | (0.284) |
|  |  |  |  |  |  |  |  |  |
| Observations | 9,264 | 9,264 |  | 4,680 | 4,680 |  | 4,584 | 4,584 |
| chi2 | 560.2 | 560.2 |  | 290.3 | 290.3 |  | 251.8 | 251.8 |
| Log Likelihood | -1405 | -1405 |  | -675.7 | -675.7 |  | -734.7 | -734.7 |

Standard errors in parentheses; *** p<0.01, ** p<0.05, * p<0.1; SD, standard deviation
